# Effectiveness of a bedaquiline, linezolid, clofazimine ‘core’ for multidrug-resistant TB

**DOI:** 10.5588/ijtldopen.24.0515

**Published:** 2025-05-12

**Authors:** C. Zeng, M.A. Hernán, L. Trevisi, S. Sauer, C.D. Mitnick, C. Hewison, M. Bastard, P. Khan, K.J. Seung, M.L. Rich, S. Law, M. Kikvidze, O. Kirakosyan, A. Miankou, P. Thit, S. Mamsa, A. Janmohamed, N. Melikyan, S. Ahmed, D. Vargas, A.B. Binegdie, K. Temirova, L. Oyewusi, K. Philippe, S.C. Vilbrun, U. Khan, H. Huerga, M.F. Franke

**Affiliations:** ^1^ Department of Global Health and Social Medicine, Harvard Medical School, Boston, MA, USA;; ^2^ CAUSALab, Departments of Epidemiology and Biostatistics, Harvard T.H. Chan School of Public Health, Boston, MA, USA;; ^3^ Partners In Health (PIH), Boston, MA, USA;; ^4^ Division of Global Health Equity, Brigham and Women’s Hospital, Boston, MA, USA;; ^5^ Medical Department, Médecins Sans Frontières (MSF), Paris, France;; ^6^ Field Epidemiology Department, Epicentre, Paris, France;; ^7^ Interactive Research and Development (IRD) Global, Singapore;; ^8^ Department of Clinical Research, Faculty of Infectious and Tropical Diseases, London School of Hygiene & Tropical Medicine, London, UK;; ^9^ MSF, Sokhumi, Georgia;; ^10^ MSF, Yerevan, Armenia;; ^11^ MSF, Minsk, Belarus;; ^12^ MSF, Yangon, Myanmar;; ^13^ Indus Hospital & Health Network (IHHN), Karachi, Pakistan;; ^14^ Interactive Research and Development, Karachi, Pakistan;; ^15^ PIH/Socios En Salud Sucursal Peru, Lima, Peru;; ^16^ Addis Ababa University College of Health Sciences, Department of Internal Medicine, Addis Ababa, Ethiopia;; ^17^ PIH, Almaty, Kazakhstan;; ^18^ PIH, Lesotho, Maseru, Lesotho;; ^19^ PIH, Port-au-Prince, Haiti;; ^20^ Haitian Group for the Study of Kaposi’s Sarcoma and Opportunistic Infections (GHESKIO), Port-au-Prince, Haiti;; ^21^ Department of Epidemiology, Harvard T.H. Chan School of Public Health, Boston, MA, USA.

**Keywords:** fluoroquinolone resistance, rifampicin resistance, target trial, inverse-probability weighting, endTB observational study, MDR-TB, tuberculosis

## Abstract

**BACKGROUND:**

Treatment outcomes may be compromised among individuals with multidrug/rifampicin-resistant TB (MDR/RR-TB) with fluoroquinolone (FQ) resistance. Among people in whom an FQ was unlikely to be effective, we compared the effectiveness of longer individualised regimens comprised of bedaquiline (Bdq) for 5–8 months, linezolid, and clofazimine to those reinforced with at least 1 Group C drug and/or longer Bdq duration.

**METHODS:**

We emulated a target trial to compare the effectiveness of initiating and remaining on the core regimen to a regimen reinforced with 1) Bdq for ≥9 months, 2) Bdq for ≥9 months, and delamanid (Dlm), 3) imipenem (Imp), 4) a second-line injectable, or 5) Bdq for ≥9 months, Dlm and Imp. We used cloning, censoring, and inverse-probability weighting to estimate the probabilities of successful treatment.

**RESULTS:**

Adjusted probabilities of successful treatment ranged from 0.75 (95% CI 0.61–0.89) to 0.84 (95% CI 0.76–0.91). Ratios of treatment success ranged from 1.01 for regimens reinforced with Bdq ≥9 months (95% CI 0.79–1.28) and Bdq ≥9 months plus Dlm (95% CI 0.81–1.31) to 1.11 for regimens reinforced with an injectable (95% CI 0.92–1.39) and Bdq ≥9 months, Dlm and Imp (95% CI 0.90–1.41).

**CONCLUSIONS:**

Some reinforced regimens had modestly higher treatment success rates, but estimates were imprecise. Additional studies of strategies for maximising treatment success among individuals with FQ resistance are needed.

The use of new and repurposed drugs, such as bedaquiline (Bdq), delamanid (Dlm), linezolid (Lzd), pretomanid (Pa), and clofazimine (Cfz), has dramatically improved the effectiveness of treatment for multidrug- or rifampicin-resistant TB (MDR/RR-TB).^1–5^ For the first time in history, the recommended treatment duration for MDR/RR-TB is as short as 6–9 months for most patients.^6–10^ However, longer 18–20 month regimens are still recommended for individuals in whom shorter regimens cannot be used (i.e., due to confirmed or suspected drug resistance and/or unavailability of drugs in the shorter regimens). Key research priorities highlighted by the WHO with regard to longer MDR/RR-TB regimens are studies on the optimal combination of drugs for patients previously treated for MDR/RR-TB, the approach to regimen design, and the optimal duration of Bdq.^6^

Patients previously treated for MDR/RR-TB are at increased risk for unfavourable treatment outcomes, in part due to a higher risk of resistance to fluoroquinolones (FQs), a cornerstone of longer individualised treatments for MDR/RR-TB.^11,12^ In this paper, we sought to address knowledge gaps about the optimal regimen for patients requiring MDR/RR-TB treatment for whom an FQ is unlikely to be effective. Specifically, we emulated a target trial to compare the effectiveness of initiating and remaining on a longer 18–20 month ‘core’ regimen comprised of Bdq for 5–8 months, Lzd, and Cfz, as compared to longer regimens that were reinforced with at least one Group C (i.e., third tier) drug and/or a longer duration of Bdq.

## METHODS

### Data resources and study population

The prospective endTB observational cohort (NCT03259269) aimed to generate evidence on the safety and effectiveness of Bdq or Dlm when used as part of a longer 18–20 month multidrug regimen for MDR/RR-TB.^13^ The cohort includes 2,788 patients from 17 countries who initiated a Bdq- or Dlm-containing regimen between April 2015 and September 2018 and consented to be enrolled. Each participant was followed according to local programme norms. Data were collected using standardised forms and entered into an electronic medical record. For this analysis, we excluded participants from the Democratic People’s Republic of Korea (DPRK) due to differences in clinical protocols and treatment (i.e., the use of shortened regimens) compared with the rest of the cohort.

### Overview of target trial emulation

Drawing causal inferences from observational data can be viewed as an attempt to emulate a hypothetical randomised pragmatic trial (i.e., a ‘target trial) using the observational dataset.^14–16^ The first step is the specification of the protocol of the target trial (including eligibility criteria, treatment strategies, treatment assignment, start and end of follow-up procedures, and outcomes). The second step is to emulate this trial using the observational dataset.^16^ The target trial framework facilitates a clear specification of the research question, transparency of assumptions, and mitigation of common biases of observational studies.^17^ Below, we describe the target trial corresponding to our research question.

### Specification of the target trial

The target trial (Table 1) would enrol, within a week of MDR/RR-TB treatment initiation, participants in whom an FQ is unlikely to be effective and Bdq, Lzd, and Cfz were likely to be effective. The likely effectiveness of a drug in a participant is established based on a susceptible drug susceptibility test (DST) or absence of prior exposure (if no DST result was available) for at least 1 month. Because there is no reliable DST for cycloserine, it is presumed not likely effective based on prior exposure only.

**Table 1.** Target trial protocol and emulation of the target trial using endTB observational cohort data.

Component	Specification	Emulation using endTB observational cohort
Eligibility criteria	Confirmed MDR/RR-TB Started Bdq within a week of treatment initiation FQ unlikely to be effective Likely effectiveness to at least Bdq, Lzd, and Cfz	Same
Treatment strategies	0 Bdq (5 to 8 months)-Lzd-Cfz 1 Bdq (≥9 months)-Lzd-Cfz 2 Bdq (≥9 months)-Lzd-Cfz-Dlm 3 Bdq (≥5 months)-Lzd-Cfz-Imp 4 Bdq (≥5 months)-Lzd-Cfz-SLI 5 Bdq (≥9 months)-Lzd-Cfz-Dlm-Imp Regimens are intended to last 18–20 months, but clinicians determine the total duration of treatment and of each individual drug, except Bdq, which is protocolised into one of three durations: ≥5 months, 5–8 months, and ≥9 months Clinician-directed Bdq interruptions of <14 days are allowable for any reason (e.g., toxicity, drug stockout) Clinically directed Bdq interruptions or drug additions for >14 days are not permitted unless they occur in response to an adverse event or newly detected resistance to a drug in the assigned treatment strategy Bdq can be reinitiated after an allowed stoppage, if indicated Drugs other than those in the assigned regimen can be included only if they are unlikely to be effective	Same
Treatment assignment	Individuals are randomly assigned a treatment strategy on day 14 and are aware of their assignment. Randomisation is stratified by the profile of likely effective drugs	Individuals are assigned to the treatment strategies that their observed data are compatible with on day 14 of treatment
Follow-up period	Follow-up starts at assignment and continues until the occurrence of an end-of-treatment outcome	Same
Outcome	Treatment success (i.e., cure or treatment completion). Individuals who die, become lost to follow-up, or in whom treatment fails would be considered to have unsuccessful treatment outcomes	Same
Causal contrast of interest	Intention-to-treat and per-protocol effects	Observational analogue of the per-protocol effect
Analysis plan	Per-protocol effect: Individuals are censored if their treatment deviates from their assigned strategy, and IP weighting is used to adjust for potential selection bias introduced by censoring. Data are pooled across strata after confirming comparable baseline risk across strata. Estimates are adjusted for baseline covariates that differ across groups. Probabilities of treatment success are compared via ratios and differences	Same, except that person clones are used

MDR/RR-TB = multidrug- or rifampicin-resistant tuberculosis; Bdq = bedaquiline; FQ = fluoroquinolone; Lzd = linezolid; Cfz = clofazimine; Dlm = delamanid; Imp = imipenem; SLI = second-line injectable; IP = inverse-probability.

Two weeks after treatment initiation and following completion of the Bdq loading phase, each eligible individual would be randomly assigned to one of six treatment strategies: 0) the core regimen (Bdq 5–8 months-Lzd-Cfz) or one of five reinforced regimens: 1) Bdq (≥9 months)-Lzd-Cfz; 2) Bdq (≥9 months)-Lzd-Cfz-Dlm; 3) Bdq (≥5 months)-Lzd-Cfz-imipenem (Imp); 4) Bdq (≥5 months)-Lzd-Cfz-second-line injectable (SLI); 5) Bdq (≥9 months)-Lzd-Cfz-Dlm-Imp. To ensure that all drugs in the reinforced regimens are likely effective in the participants assigned to them, randomisation is stratified based on the profile of likely effective drugs. For example, individuals for whom Dlm and Imp are likely effective, but an SLI is not, are placed into a stratum where the possible treatment assignments include 0, 1, 2, 3, and 5, but exclude 4.

Lzd is initiated at 600 mg daily but can be reduced to other doses if indicated (i.e., due to toxicity or intolerability). Regimens are intended to last 18 to 20 months, but clinicians determine the total duration of treatment and of each individual drug, except Bdq, which is assigned by protocol into one of three durations: ≥5 months, 5–8 months, or ≥9 months. Clinician-directed Bdq interruptions of <14 days are allowable for any reason (e.g., toxicity, drug stockout). Bdq interruptions or drug additions for >14 days are not permitted unless they occur in response to an adverse event (AE) or newly detected resistance to a drug in the assigned treatment strategy. Bdq can be reinitiated after an allowed stoppage if indicated. Drugs other than those in the assigned regimen can be included only if they are unlikely to be effective.

The outcome of interest is treatment success, defined as cure or treatment completion at the end of treatment. Death, treatment failure, and loss to follow-up are considered unsuccessful end-of-treatment (EOT) outcomes. All EOT outcomes are calculated based on WHO guidance, and the first point at which failure occurs is identified.^18,19^ For each individual, follow-up would start at assignment to a regimen (time 0) and continue each week until the end of treatment. The causal contrasts of interest are the intention-to-treat and the per-protocol effects.

### Statistical analysis of the target trial

In the intention-to-treat analysis, the probabilities of treatment success in each group can be estimated nonparametrically or via a parametric logistic regression model for the weekly probability of treatment success (the model can include baseline covariates if their distribution differs between groups). To increase precision, data can be pooled across strata, assuming a common baseline risk (this can be tested empirically by comparing the probability of the outcome across the control groups, standardising by covariates as needed). The predicted probabilities of treatment success are then compared via success ratios and differences. The 95% confidence intervals are computed using the bootstrapping method with 500 samples.

The per-protocol analysis is identical, except that individuals are censored if/when their treatments deviate from their assigned strategy for any reason other than adverse effects or acquired resistance to a drug in the assigned strategy. Specifically, individuals are censored if they have any new likely effective drugs added to their assigned strategy or if they do not follow the assigned duration of Bdq. To adjust for the potential selection bias introduced by censoring, we can incorporate inverse probability (IP) weights.^20^

### Target trial emulation

We emulated the target trial using the endTB prospective observational dataset (Table 1). Because individuals can have data compatible with more than one treatment strategy at time 0, we made one modification to the per-protocol analysis: we cloned individuals in the dataset and assigned each clone to each of the Bdq durations (i.e., Bdq ≥5 months, Bdq 5–8 months, or Bdq ≥9 months) that were compatible with their observed data at time 0. Supplementary Figure S1 shows an overview of the cloning and censoring steps. We fit an IP-weighted logistic regression model for the probability of treatment success among uncensored clones that included the following baseline covariates:^19^ treated in Georgia (yes/no), year of enrollment (continuous), low body mass index (BMI) (yes/no), sputum smear (positive/negative), sputum culture (positive/negative), and received cycloserine (Cs), although it was unlikely to be effective in the individual (yes/no). The country of Georgia was included because it had a disproportionate number of participants with regimens reinforced with an SLI.

We estimated the denominator of the stabilised IP weights using separate logistic models for the weekly probability of ‘not adding any new likely effective drugs to the baseline regimens’ and of ‘remaining on Bdq’ conditional on the baseline covariates and the following time-varying covariates: sputum smear (positive/negative) and receipt of Cs when it was unlikely to be effective in the individual (yes/no). Time was modelled using linear and quadratic terms for the week of follow-up. We estimated the numerator of the stabilised IP weights using analogous models without time-varying covariates. Supplementary Table S1 describes the calculation of IP weights for each treatment strategy. IP weights were not truncated. We used missing indicator variables to account for the small amount of missing data.

### Research ethics

The endTB observational study protocol was approved by all study countries and central ethics review committees for each consortium partner (Partners In Health, Doctors Without Borders, Epicentre, and Interactive Research and Development). Participants provided written informed consent for inclusion in the observational cohort.

## RESULTS

A total of 443 individuals were included in our analysis (Supplementary Figure S2). One-third (149/443) of the participants were female, and the median age was 35 years (interquartile range [IQR] 28–45) (Table 2). At the time of treatment initiation, the prevalence of comorbidities was 4% HIV infection, 12% diabetes mellitus or glucose intolerance, 6% hepatitis B infection, and 17% hepatitis C infection. Also, 96% had documented resistance to an FQ, 69% had bilateral disease, 75% had cavitary disease, and 59% had a positive sputum smear.

**Table 2.** Baseline characteristics of 443 individuals receiving treatment for MDR/RR-TB who were previously treated with second-line TB drugs, 2015–2018.

Characteristics	*n* (%)
Demographic characteristics	
Age, years, median [IQR] (range)	35 [28–45] (14–71)
Female	149 (34)
Marital status, married or living together	211 (48)
Substance and drug use	
Alcohol use	55 (12)
Tobacco use (*n* = 441)	142 (32)
Injection drug use (*n* = 441)	6 (1)
Non injection drug use (*n* = 439)	14 (3)
Comorbidities	
Anaemia (*n* = 439)	181 (41)
HIV infection	19 (4)
Diabetes mellitus or glucose intolerance (*n* = 442)	53 (12)
Hepatitis B virus infection (*n* = 442)	28 (6)
Hepatitis C virus infection	77 (17)
At least one comorbidity other than those above	48 (11)
TB-related characteristics	
Prior TB treatment with second-line drugs	439 (99)
Bilateral disease (*n* = 427)	296 (69)
Cavitary disease (*n* = 417)	313 (75)
Positive culture (*n* = 412)	276 (67)
Positive smear (*n* = 423)	250 (59)
Resistance profile	
MDR/RR-TB without any testing to FQ	33 (7)
MDR/RR-TB with resistance to any FQ	410 (93)
Number of not likely effective drugs included in baseline regimen, median [IQR] (range)	1 [0–2] (0–5)
Impaired functional status (limited self-care or completely disabled) (*n* = 377)	192 (51)
Body mass index <18.5 kg/m^2^ (*n* = 442)	154 (35)

IQR = interquartile range; MDR/RR-TB = multidrug- or rifampicin-resistant tuberculosis; FQ = fluoroquinolone.

Of the 443 individuals, 23% (*n* = 100) initiated a regimen containing Bdq, Lzd, and Cfz without additional likely effective drugs. The remaining individuals received one or more likely effective third-tier drugs, including Dlm (18%, *n* = 81), Imp (17%, *n* = 74), SLI (23%, *n* = 103), or Dlm and Imp together (19%, *n* = 85). Only one individual 0.2%initiated Lzd at 600 mg daily. Regimens often contained drugs that were unlikely to be effective, most commonly pyrazinamide, cycloserine, or an FQ (Supplementary Table S2). Cs, which was unlikely to be effective, was used in 65% (65/100) of participants who initiated Bdq-Lzd-Cfz and 67% (69/103) who initiated a reinforced regimen containing an injectable. In other regimens, the receipt of Cs was less frequent.

The probability of treatment success ranged from 0.75 (95% CI 0.61–0.89) for Bdq (5–8 months)-Lzd-Cfz to 0.84 (95% CI 0.76–0.91) for Bdq (≥5 months)-Lzd-Cfz-SLI (Table 3). Compared with Bdq (5–8 months)-Lzd-Cfz, the treatment success ratios ranged from 1.01 for regimens reinforced with Bdq ≥9 months (95% CI 0.79–1.28) and Bdq ≥9 months plus Dlm (95% CI 0.81–1.31) to 1.11 for regimens reinforced by a second-line injectable (95% CI 0.92–1.39) and by Bdq ≥9 months, Dlm and Imp (95% CI 0.90–1.41) (Table 3). The risk difference ranged from 0.01 for regimens reinforced with Bdq ≥9 months (95% CI –0.18 to 0.17) and Bdq ≥9 months plus Dlm (95% CI –0.16 to 0.19) to 0.08 for regimens reinforced by a second-line injectable (95% CI –0.08 to 0.23) and by Bdq ≥9 months, Dlm and Imp (95% CI –0.08 to 0.26) (Figure 1). The mean of stabilised IP weights was 1.00 (standard deviation: 0.13; range: 0.71–4.20).

**Table 3.** Adjusted probabilities and ratios of MDR/RR-TB end-of-treatment success among individuals previously treated with second-line TB drugs.*

Treatment strategy	Adjusted probability of treatment success (95% CI)	Adjusted ratio of treatment success (95% CI)
Bdq-(5–8 months)-Lzd-Cfz	0.75 (0.61–0.89)	Reference
Bdq-(≥9 months)-Lzd-Cfz	0.77 (0.64–0.87)	1.01 (0.79–1.28)
Bdq-(≥9 months)-Lzd-Cfz-Dlm	0.76 (0.65–0.87)	1.01 (0.81–1.31)
Bdq-(≥5 months)-Lzd-Cfz-Imp	0.80 (0.68–0.90)	1.06 (0.83–1.34)
Bdq-(≥5 months)-Lzd-Cfz-SLI	0.84 (0.76–0.91)	1.11 (0.92–1.39)
Bdq-(≥9 months)-Lzd-Cfz-Dlm-Imp	0.83 (0.740.91)	1.11 (0.90–1.41)

* Adjusted for baseline values of year of enrolment (i.e., 2015, 2016, 2017, 2018), treated in Georgia (yes/no), low BMI (yes/no), sputum smear (negative/positive), sputum culture (negative/positive), received cycloserine that was unlikely to be effective (yes/no), and time-varying receipt of cycloserine (yes/no), and time-varying sputum smear (negative/positive), missing indicator of sputum culture.

MDR/RR-TB = multidrug- or rifampicin-resistant tuberculosis; CI = confidence interval; Bdq = bedaquiline; Lzd = linezolid; Cfz = clofazimine; Dlm = delamanid; Imp = imipenem; SLI = second-line injectable.

**Figure.**
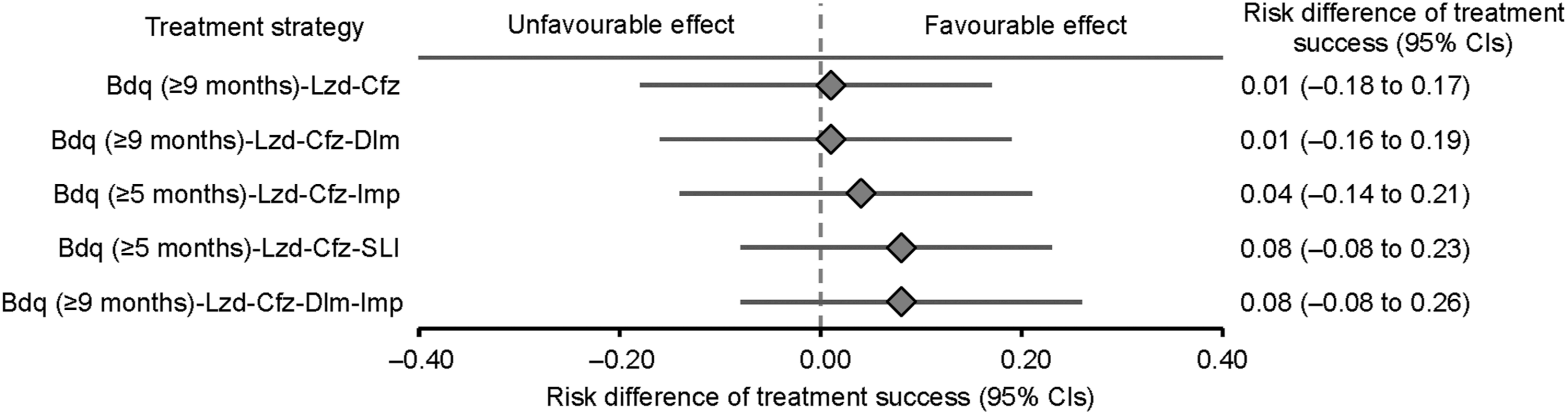
Adjusted risk differences for treatment success of reinforced regimens compared to the Bdq (5–8 months)-Lzd-Cfz core regimen. CI = confidence interval; Bdq = bedaquiline; Lzd = linezolid; Cfz = clofazimine; Dlm = delamanid; Imp = imipenem; SLI = second-line injectable.

## DISCUSSION

In a cohort of individuals with MDR/RR-TB in whom an FQ was unlikely to be effective, longer regimens containing at least Bdq for 5 months, Lzd, and Cfz yielded proportions of treatment success that were higher than historical rates,^21^ but still lower than those achieved with all oral shortened regimens for FQ-susceptible TB^4,5,9,22^ and lower than those reported in trials of all oral shortened regimens for FQ-resistant TB.^10,23^ The regimens reinforced with Dlm alone and/or a longer duration of Bdq alone were not associated with an improved frequency of treatment success compared with the Bdq (5–8 months)-Lzd-Cfz core regimen. Regimens reinforced with an injectable or Bdq ≥9 months, Dlm and Imp had modestly higher treatment success rates, but under conventional statistical criteria, anything between a 10% reduction in success and a 40% increase in success was highly compatible with the data.

Even though the present analysis did not identify specific drugs to be added to the Bdq, Lzd, and Cfz core to improve effectiveness, a regimen comprising four versus three likely effective medications has potential benefits. These include protection against acquired resistance (in case of failure or relapse) and assurance of an adequate number of effective drugs in the case of undetected resistance to one of the drugs. All WHO-endorsed regimens—long and short, for FQ-susceptible or -resistant MDR/RR-TB—use at least four drugs, with one exception.^6^ The three-drug 6-month regimen for FQ-resistant MDR/RR-TB, Bdq-Pa-Lzd (known as BPaL), was evaluated in uncontrolled studies^10,23^ and one randomised, internally controlled trial in which a very small number of participants with FQ-resistant TB received this regimen (*n* = 25).^24^ All MDR/RR-TB regimens endorsed by WHO are based on low or very low certainty evidence, and recommendations are conditional.^6,25^ While the conditionality means that recommendations are likely to change with emerging evidence, our results do not support a change in the number of drugs recommended in longer regimens for FQ-resistant MDR/RR-TB.

It is possible that reinforcing the core regimen with drugs or combinations not examined here (e.g., Pza, Cs) may improve effectiveness. Of note, the 2022 WHO guidance recommended that longer regimens for MDR/RR-TB prioritise drugs from Groups A (Bdq, Lzd, FQ) and B (Cfz and Cs).^6^ Under this guidance, the recommended longer regimen for someone with FQ-resistant TB comprises at least the four-drug regimen Bdq-Lzd-Cfz-Cs. We were unable to study this regimen because Cs—although used frequently— rarely met the definition of a likely effective drug in this cohort of individuals previously exposed to second-line drugs.

The use of a target trial framework to address this research question facilitated inference and interpretation in several regards. First, it enabled clear articulation of the research question and the treatment strategies to be compared. Second, it informed the design of an analysis that accounted for two important sources of potential bias: 1) regimens were assigned and changed based on clinical judgment, not at random, which can lead to important differences among the individuals who receive each regimen (i.e., confounding); 2) individuals who live longer can be treated longer (i.e., immortal person-time bias).^15,26^ Although both biases are important considerations in observational comparative effectiveness analyses of treatment duration, methods to account for them appropriately have been applied infrequently in TB cohorts.^27–29^

However, the target trial framework does not eliminate confounding and measurement errors. The relatively small sample size for this analysis resulted in imprecise estimates and forced us to adjust only for the strongest and most likely confounders. For example, the regimen reinforced with an injectable was disproportionately used in Georgia, and we adjusted for this site but could not adjust for all 13 sites even though regimen composition is based on national TB programme guidelines. Another limitation of the data, the imperfect classification of the likely effectiveness of drugs in an individual, reflects the reality faced by TB clinicians globally: in the absence of widely accessible, highly accurate DST testing for all second-line TB drugs, there will be uncertainty about which drugs are active in a given patient, which could bias results in unpredictable ways.

These findings highlight the challenge of discerning the impact of specific drugs added to a robust background regimen.^30,31^ Identifying small to moderate improvements in effectiveness will require large sample sizes, underscoring the importance of collaborative multisite initiatives to harmonise and pool data to fill critical evidence gaps. Future studies with larger sample sizes will also permit subgroup analyses (individuals with extensive disease may require longer treatment —and/or regimens containing more drugs— than those without extensive disease)^32,33^ and study less common events such as TB relapse (in the endTB observational study, recurrent TB rarely occurred – only 10 cases within 6 months among 1228 individuals with a successful end-of-treatment outcome and complete follow-up),^34^ safety events and person-centred outcomes, such as tolerability or preference. As MDR/RR-TB regimen effectiveness continues to improve, these outcomes may offer a way to distinguish between highly effective regimens.

In conclusion, among individuals with MDR/RR-TB in whom an FQ is unlikely to be effective due to resistance or prior exposure to the second-line treatment, we could not identify a specific strategy for reinforcing a core regimen of Bdq (5–8 months)-Lzd-Cfz with additional Group C drugs and/or a longer duration of Bdq which improved treatment effectiveness. Consequently, the WHO-recommended, non-specific strategy of using two Group A drugs and completing the minimum four-drug regimen with Group B or C drugs cannot be made more precise. Overall, these findings point to a continued need for research on how to improve end-of-treatment success in individuals in whom an FQ is unlikely to be effective.

## Supplementary Material


