# Supplementary material for: Effectiveness of a bedaquiline, linezolid, clofazimine ‘core’ for multidrug-resistant TB
Source: IJTLD Open. 2025 May 12;2(5):269–75. doi: 10.5588/ijtldopen.24.0515 (PMC12068452; doi:10.5588/ijtldopen.24.0515)
Supplement: Supplementary file 1 [file ijtldopen24-0515_supplementarydata1.docx]

# **Effectiveness of a bedaquiline, linezolid, clofazimine ‘core’ for multidrug-resistant TB**

## **Supplementary Data**

**Effectiveness of a bedaquiline, linezolid, clofazimine “core” for multidrug-resistant tuberculosis**

Chengbo Zeng^1^, Miguel A. Hernán^2^, Letizia Trevisi^1^, Sara Sauer^1^, Carole D. Mitnick^1,3,4^, Catherine Hewison^5^, Mathieu Bastard^6^, Palwasha Khan^7,8^, Kwonjune J. Seung^1,3,4^, Michael L. Rich^1,3,4^, Stephanie Law^1^, Marina Kikvidze^9^, Ohanna Kirakosyan^10^, Alexey Miankou^11^, Phone Thit^12^, Shahid Mamsa^13^, Aleeza Janmohamed^14^, Nara Melikyan^6^, Saman Ahmed^14^, Dante Vargas^15^, Amsalu Bekele Binegdie^16^, Kulbaram Temirova^17^, Lawrence Oyewusi^18^, Kerline Philippe^19^, Stalz C. Vilbrun^20^, Uzma Khan^7†^, Helena Huerga^6†^, Molly F. Franke^1,21†^, on behalf of the endTB Observational Study Team.

**Author affiliations**

1. Department of Global Health and Social Medicine, Harvard Medical School, Boston, Massachusetts, USA
2. CAUSALab, Departments of Epidemiology and Biostatistics, Harvard T.H. Chan School of Public Health, Boston, Massachusetts, USA
3. Partners In Health, Boston, Massachusetts, USA
4. Division of Global Health Equity, Brigham and Women’s Hospital, Boston, Massachusetts, USA
5. Medical Department, Médecins Sans Frontières, Paris, France
6. Field Epidemiology Department, Epicentre, Paris, France
7. Interactive Research and Development (IRD) Global, Singapore
8. Department of Clinical Research, Faculty of Infectious and Tropical Diseases, London School of Hygiene & Tropical Medicine, London, UK
9. Médecins Sans Frontières, Sokhumi, Georgia
10. Médecins Sans Frontières, Yerevan, Armenia
11. Médecins Sans Frontières, Minsk, Belarus
12. Médecins Sans Frontières, Yangon, Myanmar
13. Indus Hospital & Health Network (IHHN), Karachi, Pakistan
14. Interactive Research and Development, Karachi, Pakistan
15. Partners In Health/ Socios En Salud Sucursal Peru, Lima, Peru
16. Addis Ababa University College of Health Sciences, Department of Internal Medicine, Addis Ababa, Ethiopia
17. Partners In Health, Almaty, Kazakhstan
18. Partners In Health, Lesotho, Maseru, Lesotho
19. Partners In Health, Port-au-Prince, Haiti
20. Haitian Group for the Study of Kaposi’s Sarcoma and Opportunistic Infections (GHESKIO), Port-au-Prince, Haiti
21. Department of Epidemiology, Harvard T.H. Chan School of Public Health, Boston, Massachusetts, USA

†: These authors contributed equally to this work.

**Corresponding author**: Molly F. Franke, Department of Global Health and Social Medicine, Harvard Medical School, 641 Huntington Avenue, Boston, Massachusetts, 02115. Email: [molly_franke@hms.harvard.edu](mailto:molly_franke@hms.harvard.edu), Phone: +1 (617) 432-5224, Fax: +1 (617) 432-2565.

**Abbreviations:**

Bdq: Bedaquiline

Cfz: Clofazimine

Cs: Cycloserine

Dlm: Delamanid

Imp: Imipenem

Lzd: Linezolid

PAS: *p*-aminosalicylic acid

SLI: Second-line injectable

DPRK: Democratic People’s Republic of Korea

DST: drug susceptibility test

MDR/RR-TB: Multidrug- or rifampicin- resistant tuberculosis

EOT: End-of-treatment

**Table S1. Probabilities of being uncensored for each bedaquiline duration strategy and follow-up period**

| **Bdq duration strategy** | **Probabilities of being uncensored** | | | |
| --- | --- | --- | --- | --- |
|  | **0 < *t* < 5-month** | **5-month < *t* ≤ 8-month** | **9-month** | ***t* > 9-month** |
| **At least 5 months** |  | | | |
| Bdq, Lzd, Cfz, Imp | 1*Prob. no added drugs | 1*Prob. no added drugs | 1*Prob. no added drugs | 1*Prob. no added drugs |
| Bdq, Lzd, Cfz, SLI | 1*Prob. no added drugs | 1*Prob. no added drugs | 1*Prob. no added drugs | 1*Prob. no added drugs |
| **5 to 8 months** |  |  |  |  |
| Bdq, Lzd, Cfz | 1*Prob. no added drugs | 1*Prob. no added drugs | (1-Prob. on Bdq for those who had never stopped Bdq before^‖^)* Prob. no added drugs | 1*Prob. no added drugs |
| **At least 9 months** |  | | | |
| Bdq, Lzd, Cfz | 1*Prob. no added drugs | Prob. on Bdq*Prob. no added drugs | 1*Prob. no added drugs | 1*Prob. no added drugs |
| Bdq, Lzd, Cfz, Dlm | 1*Prob. no added drugs | Prob. on Bdq*Prob. no added drugs | 1*Prob. no added drugs | 1*Prob. no added drugs |
| Bdq, Lzd, Cfz, Dlm, Imp | 1*Prob. no added drugs | Prob. on Bdq*Prob. no added drugs | 1*Prob. no added drugs | 1*Prob. no added drugs |

Abbreviations: Prob: Probability. *t*: Time. ‖: For other individuals, it is “1*Prob. no added drugs”.

The probability of being uncensored is calculated for each week. At time 0, the probability of being uncensored ($p_{0}$) is 1. Subsequently, for each week *i*, the overall probability of being uncensored ($P_{i}$) is the cumulative product of the conditional probabilities of being uncensored in each week up to and including week *i*, i.e. $p_{0}$,…, $p_{i}$ (Table E2). The formula for calculating $P_{i}$ is shown in Formula 1:

$$P_{i}=P_{i-1}\cdot p_{i}=\left( \prod_{t=0}^{i-1} p_{t} \right)\cdot p_{i} (Formula 1)$$

For each week, the weight ($W_{i}$) is calculated as the inverse of the probability of being uncensored ($P_{i}$) (Formula 2). The final weight at the end of treatment was retained for each individual and adjusted in the analysis.

$$W_{i}=\frac{1}{P_{i}} (Formula 2)$$

**Table S2. Inclusion of drugs not likely to be effective in baseline (day 14) MDR/RR-TB regimens**

| **Drug** | **Overall**  **(N=443)**  **n (%)** | **Bdq-Lzd-Cfz**  **(N=100)**  **n (%)** | **Bdq-Lzd-Cfz-Dlm**  **(N=81)**  **n (%)** | **Bdq-Lzd-Cfz-Imp**  **(N=74)**  **n (%)** | **Bdq-Lzd-Cfz-SLI**  **(N=103)**  **n (%)** | **Bdq-Lzd-Cfz-Dlm-Imp**  **(N=85)**  **n (%)** |
| --- | --- | --- | --- | --- | --- | --- |
| Isoniazid | 1 (0) | 0 (0) | 0 (0) | 0 (0) | 1 (1) | 0 (0) |
| Ethambutol | 12 (3) | 7 (7) | 2 (2) | 0 (0) | 3 (3) | 0 (0) |
| Pyrazinamide | 164 (37) | 64 (64) | 32 (40) | 20 (27) | 43 (42) | 5 (6) |
| Amikacin | 7 (2) | 5 (5) | 1 (1) | 1 (1) | NA | 0 (0) |
| Kanamycin | 13 (3) | 11 (11) | 1 (1) | 1 (1) | NA | 0 (0) |
| Capromycin | 16 (4) | 15 (15) | 1 (1) | 0 (0) | NA | 0 (0) |
| Levofloxacin | 48 (11) | 21 (21) | 3 (4) | 7 (9) | 17 (17) | 0 (0) |
| Moxifloxacin | 90 (20) | 38 (38) | 4 (5) | 21 (28) | 26 (25) | 1 (1) |
| Cycloserine | 191 (43) | 65 (65) | 25 (31) | 27 (36) | 69 (67) | 5 (6) |
| PAS | 82 (19) | 25 (25) | 3 (4) | 21 (28) | 33 (32) | 0 (0) |
| Imipenem | 20 (5) | 2 (2) | 17 (21) | NA | 1 (1) | NA |
| Ethionomide / Prothionamide | 57 (13) | 30 (30) | 3 (4) | 2 (3) | 21 (20) | 1 (1) |

**Table S3. Prevalence of baseline bilateral disease, cavitary disease and sputum smear positivity, by regimen**

| **Treatment strategy** | **Bilateral disease**  **(N = 427)**  **n (%)** | **Cavitary disease**  **(N = 417)**  **n (%)** | **Smear positive**  **(N = 423)**  **n (%)** |
| --- | --- | --- | --- |
| Bdq-Lzd-Cfz | 67 (71) | 63 (71) | 48 (51) |
| Bdq-Lzd-Cfz-Dlm | 54 (68) | 66 (84) | 50 (63) |
| Bdq-Lzd-Cfz-Imp | 56 (81) | 43 (64) | 49 (70) |
| Bdq-Lzd-Cfz-SLI | 58 (58) | 66 (67) | 48 (48) |
| Bdq-Lzd-Cfz-Dlm-Imp | 61 (73) | 75 (90) | 55 (69) |

*443 individuals were included in this analysis. The N shown in each header reflects the number of participants with data for each variable.

**Table S4. Reasons a fluoroquinolone was considered unlikely to be effective at baseline (day 14), by regimen received (N=443)**

| **Treatment strategy** | **Criteria for no likely effectiveness** | |
| --- | --- | --- |
|  | **FQ resistance**  **n (%)** | **Prior FQ exposure for >1 month and no FQ DST* result**  **n (%)** |
| Bdq-Lzd-Cfz (N=100) | 80 (80) | 20 (20) |
| Bdq-Lzd-Cfz-Dlm (N=81) | 76 (94) | 5 (6) |
| Bdq-Lzd-Cfz-Imp (N=74) | 71 (96) | 3 (4) |
| Bdq-Lzd-Cfz-SLI (N=103) | 100 (97) | 3 (3) |
| Bdq-Lzd-Cfz-Dlm-Imp (N=85) | 83 (98) | 2 (2) |

**Table S5. Frequency of drug additions due to adverse events or newly detected resistance (N=443)***

| **Treatment strategy** | **Reason for drug addition** | |
| --- | --- | --- |
|  | **Adverse events**  **n (%)** | **Drug resistance**  **n (%)** |
| Bdq-Lzd-Cfz (N=100) | 12 (12) | 0 (0) |
| Bdq-Lzd-Cfz-Dlm (N=81) | 2 (2) | 0 (0) |
| Bdq-Lzd-Cfz-Imp (N=74) | 15 (20) | 1 (1) |
| Bdq-Lzd-Cfz-SLI (N=103) | 15 (15) | 1 (1) |
| Bdq-Lzd-Cfz-Dlm-Imp (N=85) | 1 (1) | 0 (0) |

*These were not considered censoring events


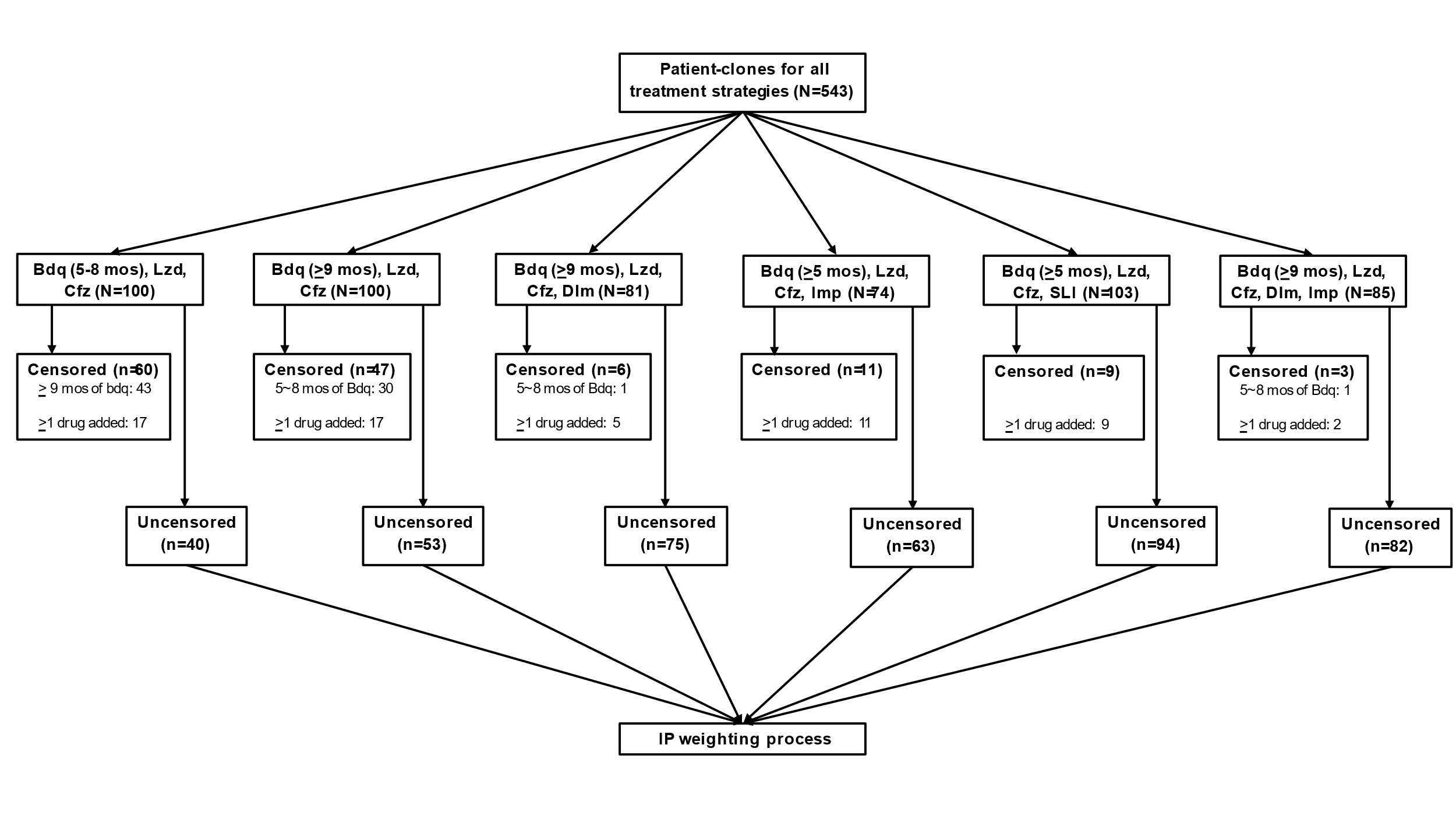


**Figure S1.** Cloning, censoring, and inverse probability weighting processes for each treatment strategy

Clones were censored if their treatment deviated from their assigned strategy; the uncensored clones were used to calculate the inverse probability weights.

**Patients enrolled in the endTB observational cohort between April 2015 to September 2018** (***N*=2,788)**

**Excluded (*N*=2,345)**

- 155 from DPRK
- 24 not MDR/RR-TB
- 376 who did not start a Bdq within one-week of treatment initiation
- 985 in whom fluoroquinolone likely to be effective
- 788 did not initiate a regimen of interest by the end of week 2
- 17 EOT outcome not evaluated

**Analytic sample (*N*=443)**

Bdq-Lzd-Cfz (*n*=100)

Bdq-Lzd-Cfz-Dlm (*n*=81)

Bdq-Lzd-Cfz-Imp (*n*=74)

Bdq-Lzd-Cfz-SLI (*n*=103)

Bdq-Lzd-Cfz-Dlm-Imp (*n*=85)

**Figure S2.** Inclusion flowchart of endTB Observational Study participants included in the present analysis
